# *Nylanderia deceptrix* sp. n., a new species of obligately socially parasitic formicine ant (Hymenoptera, Formicidae)

**DOI:** 10.3897/zookeys.552.6475

**Published:** 2016-01-13

**Authors:** Steven J. Messer, Stefan P. Cover, John S. LaPolla

**Affiliations:** 1 Department of Biological Sciences, Towson University, Towson, MD 21252 USA; 2 Department of Entomology, Museum of Comparative Zoology, Harvard University, Cambridge MA 02138 USA; 3 Department of Biology, University of Rochester, Rochester, NY 14627 USA (current address)

**Keywords:** Ants, Formicinae, Nearctic, New Species, Social Parasitism

## Abstract

Obligately socially parasitic ants are social parasites that typically lack the sterile worker caste, and depend on the host species for survival and brood care. The genus *Nylanderia* has over 130 described species and subspecies, none of which, until this study, were known social parasites. Here we describe the first social parasite known in the genus, *Nylanderia deceptrix*. Aspects of the biology of the host species, *Nylanderia parvula* (Mayr 1870), and *Nylanderia deceptrix* are examined. The data from both the host and the parasite species are combined to better understand the host-parasite relationship.

## Introduction

Social parasitism consists of an array of fascinating life history strategies that are expressed in several different ways among the ants (Nonacs and Tobin 1992, Bekkevold and Boomsma 2000, Buschinger 2009, Rabeling and Bacci Jr. 2010). Among the socially parasitic ants are those that are obligate social parasites. Obligately socially parasitic ants are characterized by a combination of life history traits where one ant species parasitizes another free-living ant species and relies on the host species for brood care and nourishment (Wilson 1971, Hölldobler and Wilson 1990, Buschinger 2009). For most ants the “typical” colony structure is one where there are one or more queens responsible for egg-laying and workers responsible for colony functions related to colony growth and development (Fischman et al. 2011). Conversely, obligate social parasites insert themselves into the colonies of other species, live with the host workers and possibly queen(s), and have their brood, which is usually only reproductives, reared by the host workers (Bekkevold and Boomsma 2000, Maschwitz et al. 2000). Of the over 13,000 described ant species, obligate social parasites are seemingly rare with about 80 known species displaying this life history strategy (Buschinger 1990, Mardulyn et al. 2014). The origin of socially parasitic ants has been of interest to myrmecologists for over a century. Emery’s Rule, which states social parasites tend to be closely related to their host species, was one of the earliest observations regarding the evolution of social parasitism in ants (Emery 1909). It can be expressed in either loose (likely closely related, but parasite and host are not sister taxa) or strict (parasite and host are sister taxa) forms. Examination of Emery’s Rule in the strict sense has provided evidence for sympatric speciation among obligate social parasites such as in *Myrmica* (Leppänen et al. 2015) and *Mycoceperus* (Rabeling et al. 2014).

The Nearctic *Nylanderia* currently consists of 14 native and 5 introduced species ranging from southern Canada to central Mexico (Kallal and LaPolla 2012). Most *Nylanderia* species around the world appear to nest in leaf litter and rotting wood (LaPolla et al. 2010), but there are habitat specialists in the Nearctic as well, such as the white sand nesting *Nylanderia phantasma* (Trager 1984) and the acorn inhabiting *Nylanderia querna* (Kallal and LaPolla 2012). All Nearctic *Nylanderia* overwinter their reproductives, which then emerge in the spring and early summer (Trager 1984, Kallal and LaPolla 2012).

Until recently *Nylanderia* species were all thought to display the “typical” colony life history discussed above, and social parasites were unknown. This changed, however, when one of us (SPC) discovered an unusual (only known from winged queens and wingless males), new *Nylanderia* species in Myles Standish State Forest in Massachusetts (USA) seemingly living in the colonies of *Nylanderia parvula* (Mayr 1870). This study is about that unusual *Nylanderia*, which we here describe as a new, obligately socially parasitic species, *Nylanderia deceptrix*, sp. n. Given that so little is known about obligately socially parasitic ant biology, the study of any obligately socially parasitic ant can provide valuable insights into this interesting biological phenomenon. Aspects of the biology of the host species, *Nylanderia parvula* (Mayr 1870), and *Nylanderia deceptrix* are examined with the hope of shedding light on how this example obligate social parasitism is expressed within *Nylanderia*.

## Materials and methods

**Figure 1.**
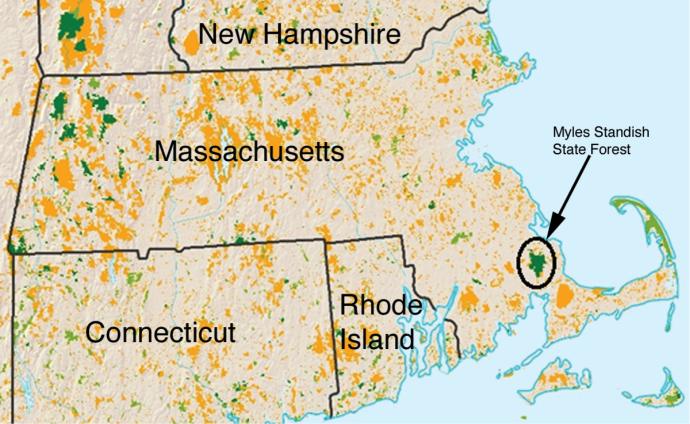
Land conservation map of Massachusetts (orange and green represent protected areas), with the location of Myles Standish State Forest indicated. Modified from http://files.usmre.com/175/MA%20Map%202009.

*Field Site*, *sampling and rearing conditions*: The field site for this study was Myles Standish State Forest in southeastern Massachusetts, the only known location of *Nylanderia deceptrix* (Fig. 1). The forest is part of the Atlantic coast pine-barrens system that stretches through the northeastern United States, including New York, New Jersey and Massachusetts (Dinerstein et al. 2015). The forest itself is open canopy, largely composed of pitch pine (*Pinus rigida*), bear/scrub oak (*Quercus ilicifloia*), and very sandy soil. Previous fieldwork had found a high density of *Nylanderia parvula* colonies in Myles Standish State Forest with the obligately socially parasitic species being collected from several colonies on at least three different collecting trips prior to this study.

Within Myles Standish State Forest collections were along Southeast Line Road (41°49.12'N, 70°39.75'W, elev. 31 m) a sandy horse trail, in June, July and September of 2013 and May, June, July and September of 2014. During these trips, colonies were excavated, and then collected and/or observed for data collection purposes described below.

Whole colonies were collected in order to determine the general population size and temporal changes within colonies of *Nylanderia parvula*. Entire colonies were excavated using a shovel and trowel, digging in the area around active nest entrances and following active chambers until observed activity ended and no part of the colony remained. Each excavated colony was examined for the presence of obligate social parasites. A minimum of eight colonies were collected and preserved for each month (May n=12, June n=10, July n=13, and September n=19 between June 2013-September 2014) for population census analysis, and the GPS coordinates of each colony was recorded. Sampled colonies were stored in large Ziploc bags, preserved in 95% ethanol and stored in a -23°C freezer until sorting. Individual ants were separated from the soil samples manually by hand and stored in vials containing 95% ethanol in a -23°C freezer. The pupal brood from each colony was determined to species (either *Nylanderia parvula* or *Nylanderia deceptrix*) by measuring queen pupae (*Nylanderia deceptrix* 2.73-3.20 mm, n=31 and *Nylanderia parvula* 3.31-3.89 mm, n=84) and wing buds on male pupae (*Nylanderia parvula* is fully winged and *Nylanderia deceptrix* has highly reduced wing buds). Eleven colonies sampled from September 2013 were removed from analysis due to lack of whole colony collection.

Colonies taken for laboratory observation had as many individuals collected as possible via aspiration and housed temporarily in a plaster bottom nest box. In the lab live colonies were transferred to larger nest boxes composed of a nesting area and a foraging area where their food was located. The nesting area had a plaster bottom that was used to maintain moisture levels within the nest to prevent desiccation of the ants within. The colonies were fed a mixture of agar, water, egg, honey and a crushed vitamin mineral capsule. This mixture was placed inside a small cap in the foraging area and was monitored to ensure mold did not form in the food and if mold was found the food was replaced. Colonies were given 5 ml of water every 2–3 days to avoid desiccation. All gaps between tubes and the nest boxes were sealed with a silicone sealant to prevent any individuals from escaping the nest box.

*Reproductive cycle*: For each colony of *Nylanderia parvula* collected (completed as explained above for a total of n= 43) the number of individuals in each caste and developmental stage was determined: alate queens, dealate queens, males, workers, larvae (1^st^–4^th^ instars) and pupae. Population census data was also compared across seasons to elucidate any temporal changes within colonies. These seasonal difference analyses included: average number of alates and average brood count.

*Taxonomic description of Nylanderia deceptrix*: All material examined was gathered from field sampling at Myles Standish State Forest (locality as specified above). Specimens of *Nylanderia deceptrix* were collected between 6 June 2013 and 16 September 2014, and preserved in 95% ethanol, and then mounted for morphological study.

Measurements were undertaken using a Leica MZ16 dissecting microscope and an ocular micrometer. Measurement terminology, abbreviations and definitions follow LaPolla et al. (2011) and Kallal and LaPolla (2012):

EL
 (Eye Length): maximum length of compound eye in full-face view.

GL
 (Gaster Length): the length of the gaster in lateral view from the anteriormost point of the first gastral segment (third abdominal segment) to the posteriormost point (in males this included through the posterior end of parameres).

HL
 (Head Length): the length of the head proper, excluding the mandibles; measured in full-face view from the midpoint of the anterior clypeal margin to a line drawn across the posterior margin from its highest points (to accommodate species where the posterior margin is concave).

HW
 (Head Width): the maximum width of the head in full-face view (in males, portion of the eyes that extends past the lateral margins of the head is included).

MMC
 (Mesonotal Macrosetae Count): the number of erect macrosetae on mesonotum to one side of sagittal plane.

MtMC
 (Metanotal Macrosetae Count): the number of erect macrosetae on metanotum to one side of sagittal plane.

MW
 (Mesonotal Width): the maximum width of the mesonotum in dorsal view.

PW
 (Pronotal Width): the maximum width of the pronotum in dorsal view.

PDH
 (Propodeum Height): height of the propodeum as measured in lateral view from the base of the metapleuron to the maximum height of the propodeum.

PFL
 (Profemur Length): the length of the profemur from its margin with the coxa to its margin with the tibia.

PFW
 (Profemur Width): the maximum width of the profemur.

PL
 (Paramere Length): the maximum length of the paramere.

PMC
 (Pronotal Macrosetal Count): the number of erect macrosetae on pronotum to one side of sagittal plane.

SL
 (Scape Length): the maximum length of the antennal scape excluding the condylar bulb.

SMC
 (Scape Macrosetal Count): the number of erect macrosetae on the scape visible in full frontal view.

TL
 (Total Length): HL+WL+GL


WL
 (Weber’s Length): in lateral view, the distance from the posteriormost border of the metapleural lobe to the anteriormost border of the pronotum, excluding the neck.

CI
 (Cephalic Index): (HW/HL) × 100


FI
 (Profemur Index): (FW/FL) × 100


REL
 (Relative Eye Index): (EL/HL) × 100


SI
 (Scape Index): (SL/HW) × 100


Each measurement was recorded to the nearest 0.001 mm and rounded to the nearest 0.01 mm. A total of 10 queens from six different colonies and five males from a single nest were used for the body measurement data. Color images were taken using a JVC KY-F75 digital camera and Syncroscopy Auto-Montage (v 5.0) software.

The male’s 9^th^ gastral sternite and penis valve were dissected under a Leica MZ16 dissecting microscope. The male’s gaster was placed in a potassium hydroxide (KOH) solution to dissolve/weaken any connective tissues and then the sternite and penis valve were separated from the gaster. The sternite and penis valves were dyed with double stain, slide mounted in glycerin and drawn using an ocular grid with a Leica DM2500 light microscope.

*Prevalence of host species and parasitism rate*: Nest entrance density for *Nylanderia parvula* was determined by counting the number of nest entrances found within four 50 m × 0.5 m transects and dividing the number found by the area (Kaspari et al. 2000, Abbott 2005, Braschler 2005). Transects were laid out along the edge of the trail that served as the study site. Once counted, a nest entrance was marked by a flag to ensure that a nest entrance was not counted twice in the same transect. One of the 50 m transects intersected with a trail, as a result the length of the transect that was intersecting the trail was excluded, resulting in two smaller transects measuring 14 m × 0.5 m and 25 m × 0.5 m.

The parasitism rate of *Nylanderia deceptrix* was determined by excavating 356 *Nylanderia parvula* colonies over the course of two collecting seasons (as stated above). In excavating to determine if *Nylanderia deceptrix* was present, a colony was excavated using a shovel and the sand containing the colony was visually inspected for *Nylanderia deceptrix*. The number of host colonies containing *Nylanderia deceptrix* (n=9) was then divided by the total number of *Nylanderia parvula* nests excavated (n=356) to calculate the parasitism rate.

*Flight and dispersal*: Morphological calculations for forewing length and Weber’s length were made and compared against each other. The forewing length (FWL) (maximum length of the forewing from mesosomal attachment to wingtip) and Weber’s length (WL) were measured for *Nylanderia deceptrix* (n=22) and the following other Nearctic *Nylanderia* species that are known to fly: *Nylanderia arenivaga* (Wheeler 1905) (n=1), *Nylanderia austroccidua* (Trager 1984) (n=1), *Nylanderia concinna* (Trager 1984) (n=3), *Nylanderia faisonensis* (Forel 1922) (n=9), *Nylanderia parvula* (n=20), *Nylanderia phantasma* (n=1), *Nylanderia querna* (n=5), *Nylanderia terricola* (Buckley 1866) (n=1), *Nylanderia vividula* (Nylander 1846) (n=13), and *Nylanderia wojciki* (Trager 1984) (n=10). For *Nylanderia deceptrix* and *Nylanderia parvula* the measurements converted to a ratio (FWL:WL), and a Student’s t-test was used to determine if there was a significant difference between the proportionate size of forewings to Weber’s length between the two species. This was done specifically to see if the wings of *Nylanderia deceptrix* were proportionally smaller than the wings of *Nylanderia parvula*, and potentially link reduced wing size to their dispersal method. All species measurements were plotted on a scatter plot for comparison.

To determine whether or not *Nylanderia deceptrix* queens could fly (males are wingless), individual queens were tested by allowing them to walk to the tip of a pencil to see if they would fly off (Rabeling and Bacci Jr. 2010). Additionally queens were dropped to see if they would fly once put into free-fall. The pencil test was conducted with five individuals with five trials each and the drop test on two individuals with two trials each.

*Aggression behavior*: Pairings for aggression tests consisted of the following combinations (all workers are of *Nylanderia parvula*; parasitized refers to coming from a colony that had *Nylanderia deceptrix* queens within it): not parasitized colony worker-foreign not parasitized colony worker (control) (n=6), parasitized colony worker-not parasitized colony worker (n=5), *Nylanderia deceptrix* queen-not parasitized colony worker (n=2), *Nylanderia deceptrix* queen-not parasitized colony *Nylanderia parvula* queen (n=16), parasitized colony worker-parasitized colony worker (n=3), and *Nylanderia deceptrix* queen-foreign parasitized colony worker (n=2). Assessment of the aggression level followed a slightly modified scale used by Abbott (2005): 1=antennation then tolerance; 2=prolonged antennation (>5 seconds) followed by tolerance; 3=rapid flight or brief gaster flexion for chemical defense; 4=brief aggression consisting of biting legs, antennae, or other body parts followed by avoidance and flight; 5=prolonged fight between individuals, potentially to the death. The number of trials for each combination varied based on the available number of individuals/colony at the time of testing.

**Figures 2–11.**
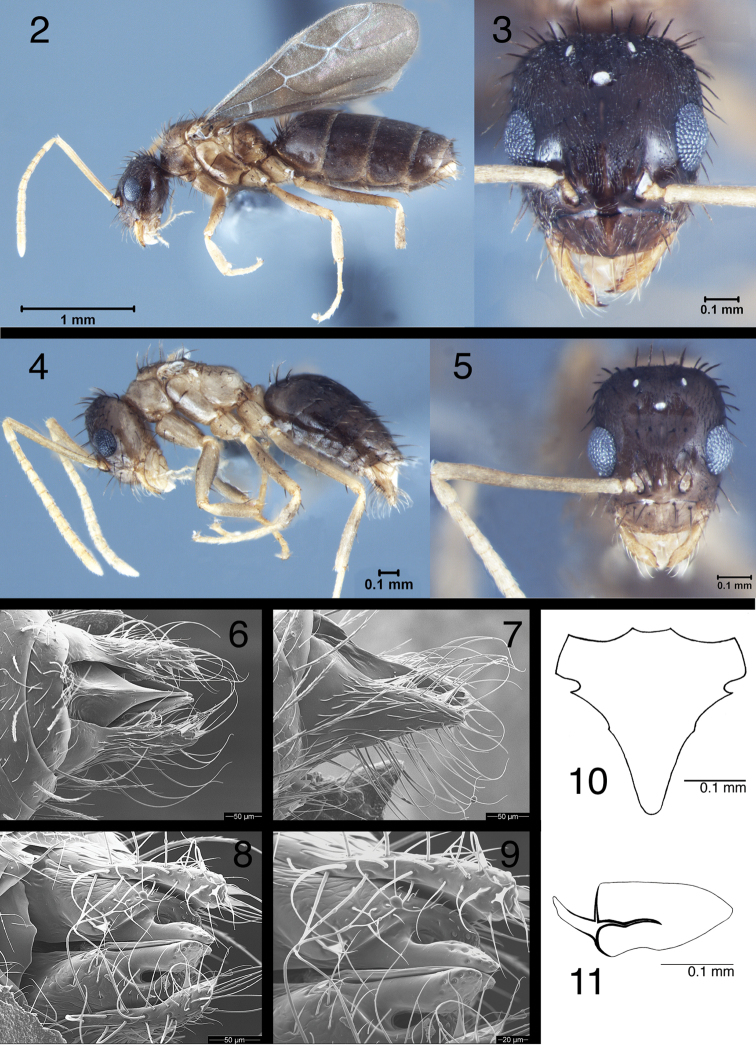
*Nylanderia deceptrix* (queen USNMENT00755074; male **4, 5** USNMENT00755083; male **6–11** USNMENT00755073): **2** queen in lateral view **3** queen head in full-frontal view **4** male in lateral view **5** male head in full-frontal view **6–9** male genitalia in dorsal, lateral, and ventral view, and ventral view close-up of digitus and cuspis **10** male 9^th^ sternite **11** penis valve (ectal view).

Introduction tests were also conducted to observe behaviors at the colony level in response to foreign individuals being introduced. Introduced individuals were placed into the foraging area of colonies (see Fig. 2) and observed. Combinations for the introduction tests consisted of the following (all workers are of *Nylanderia parvula*): not parasitized nest worker-foreign not parasitized nest (control) (n=5), *Nylanderia deceptrix* queen-foreign parasitized nest (n=3), parasitized nest worker-foreign parasitized nest (n=3), *Nylanderia deceptrix* queen-not parasitized nest (n=4), parasitized nest worker-not parasitized nest (n=5), and not parasitized nest worker-parasitized nest (n=4). The introduction tests were scored on the same scale as the aggression tests. These tests allowed for any behaviors that occur within or around the nest to be observed that may have been missed due to the inability to see within the nest during field observations (Heinze 1989, Johnson 1994).

## Results

### Nylanderia deceptrix sp. n.

Taxon classificationAnimaliaHymenopteraFormicidae

http://zoobank.org/B5A11117-4638-4B6D-9908-6208C05CF558

Figs 2 , 3 (queen); 4–11 (male)

#### Holotype

**queen**, USA. Massachusetts: Plymouth County: Myles Standish State Forest; Southeast Line Road; 41°49.12'N, 70°39.75'W; elev. 31 m; in *Nylanderia parvula* nest; 06 June 2013 (S. Messer) (MCZC); 10 paratype queens and 7 paratype males same locality information as holotype except different collection dates (MCZC and USNM).

#### Diagnosis.

*Queen*: smallest of Nearctic *Nylanderia* (TL less than 3.5 mm); mesosoma color mottled with areas of lighter and darker brown to yellowish-brown; *Male*: very small, nonfunctional wings present.

QUEEN. *Measurements* (*n*=*10*): TL: 2.91–3.40; HW: 0.55–0.63; HL: 0.58–0.69; EL: 0.22–0.24; SL: 0.73–0.78; MW: 0.52–0.57; PW: 0.55–0.67; WL: 0.99–1.07; GL: 1.24–1.69; PDH: 0.35–0.42; PFL: 0.67–0.72; PFW: 0.15–0.17; SMC: 0–3; PMC: 4–5; MMC: 21–27; MtMC: 3–4.

*Indices*: CI: 92–97; REL: 33–37; SI: 121–130; FI: 21–24.

Overall brown to yellowish-brown; head and gaster darker brown with generally lighter mesosoma; mesosoma color mottled with areas of lighter and darker brown to yellowish-brown; antennae, mandibles and legs yellow; body covered with dense pubescence; macrosetae dark brown but usually with lighter yellowish-brown tips. Eyes bulge slightly beyond head outline in full-frontal view; three prominent ocelli present. Scapes long; yellow; exceed posterior margin of the head by the length of first 3 funicular segments; scapes with dense pubescence and sometimes with up to three short standing macrosetae, but often with none. Head with abundant macrosetae and layer of pubescence; slightly longer than broad; becoming slightly wider at posterior of head. Mesosoma covered with erect macrosetae and pubescence; most macrosetae on mesonotum and metanotum show strong curvature. Gaster covered in pubescence and a large cluster of macrosetae on first gastral tergite.

MALE. *Measurements* (*n*=*5*): TL: 1.91–2.05; HW: 0.45–0.46; HL: 0.48–0.53; EL: 0.17–0.18; SL:0.57–0.59; MW: 0.28–0.32; PW: 0.37–0.39; WL: 0.66–0.69; GL: 0.74–0.88; PDH: 0.24–0.26; PFL: 0.52–0.54; PFW: 0.11–0.13; PL: 0.20–0.24; SMC: 0; PMC: 0; MMC: 7–12; MtMC: 1–2.

*Indices*: CI: 88–97; REL: 34–36; SI: 125–127; FI: 22–25.

Overall color brown to brownish-yellow; head and gaster darker brown with generally lighter mesosoma; antennae, mandibles, legs, and parameres yellow; body covered with dense pubescence; macrosetae dark brown but usually with lighter yellowish-brown tips; cuticular surface dull, covered in a dense layer of appressed setae. Head longer than broad; eyes large and bulging beyond head outline in full-frontal view; three prominent ocelli present; scapes long, exceeding posterior margin of the head by length of first 3 funicular segments; scapes absent of macrosetae and with a dense layer of pubescence; clypeus roughly rectangular, with anterior margin emarginated; mandible broad, with 4 teeth; all but apical tooth are weakly developed; apical tooth distinct, curves in toward body midline. Mesosoma relatively small; very small nonfunctional wings present; mesosoma covered in pubescence, with erect setae of varying lengths dorsally and on legs. Pronotum collar-like; mesonotum offset from pronotum at sulcus; mesonotum rises sharply above height of pronotum; mesonotum flat dorsally with many erect setae of varying lengths; marcosetae on mesonotum and metanotum show strong curvature of about 90°; propodeum indistinct from remainder of mesosoma, but with steep declivity; petiole short, triangular, upright, with posterior face only slightly longer than anterior face. Gaster with a dense layer of pubescence and erect setae; parameres especially setose; parameres roughly triangular, turning slightly mesad posteriorly; long setae extend off of parameres; cuspi small and tubular, reaching digiti dorsally; digiti weakly anvil-shaped, with poorly developed point directed ventrally; volsellar lobes flat, slightly indented relative to digital margin.

#### Etymology.

The species epithet *deceptrix* (Latin = deceiver) is attributed to the parasitic lifestyle, deceiving the host to allow cohabitation.

#### Notes.

*Nylanderia deceptrix* can be identified from other Nearctic species because it has the smallest queens of all Nearctic *Nylanderia*, ranging between 2.91–3.40 mm (Trager 1984, Kallal and LaPolla 2012). Compared to other Nearctic species with no macrosetae on the scape such as *Nylanderia parvula* and *Nylanderia trageri* (Kallal and LaPolla 2012), *Nylanderia deceptrix* is the only species with queens showing bicoloration, with the head and gaster being darker in color than the mesosoma. Additionally the queens have a mottled coloration on the mesosoma with areas of darker brown and yellow-brown. *Nylanderia deceptrix* males are currently the only Nearctic *Nylanderia* to display highly reduced wings. The male parameres display dense and very long macrosetae compared to those of other Nearctic species. The digitus displays a narrower area towards the base of the structure that expands towards the tip and ends with a narrow point. The end of the digitus also has distinct foveolate (pitted) sculpturing. The head of both the queen and the male are worker-like in overall appearance (except for the presence of distinct, large ocelli; never strongly developed in workers), and are longer than wide, whereas *Nylanderia* reproductives, especially queens, typically have wider than long heads. Additionally, *Nylanderia* queens usually have heads covered in dense pubescence, and this is not the case in *Nylanderia deceptrix*.

#### Prevalence of host species and parasitism rate.

Across the seven transects, the average *Nylanderia parvula* nest entrance density was 2.35 nest entrances/m^2^ (SD=0.15), ranging from 1.64–2.64 nest entrances/m^2^ for the individual transects. Transect 1 was excluded from all calculations because of inexperience in locating nest entrances and insufficient surveying effort resulting in a density 81.3% less than the average across all other transects.

In total, 356 *Nylanderia parvula* colonies were excavated and checked for the presence of *Nylanderia deceptrix*. Of those 356 colonies, nine had *Nylanderia deceptrix* present, resulting in a parasitism rate of 2.53%. The number of *Nylanderia deceptrix* queens found in a single colony ranged from 1–8 per colony. *Nylanderia deceptrix* males were only found in one of the nine parasitized colonies, and contained a total of nine males. *Nylanderia deceptrix* brood were found in two of the nine parasitized colonies. One colony contained only a single *Nylanderia deceptrix* queen pupa. The range for *Nylanderia parvula* pupal length was 3.31–3.89 mm (n=84) and the range for *Nylanderia deceptrix* pupal length was 2.73–3.20 mm (n=30). On the other end of the spectrum one colony contained 74 *Nylanderia deceptrix* queen pupae and 4 *Nylanderia deceptrix* male pupae (male *Nylanderia deceptrix* pupae could be determined by highly reduced wing buds and the presence of genitalia).

#### Reproductive cycle.

**Figure 12.**
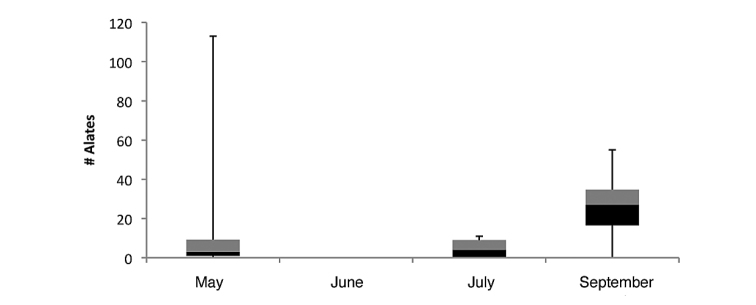
Box-and-Whisker plot of within colony *Nylanderia parvula* alate reproductive counts from May, June, July and September.

**Figure 13.**
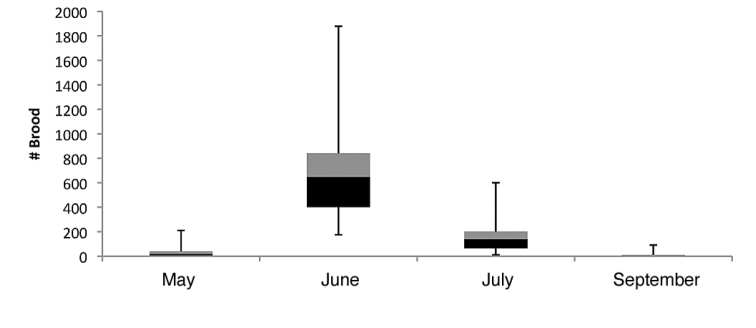
Box-and-Whisker plot of within colony *Nylanderia parvula* brood counts from May, June, July and September.

All colonies that were found to have dealate queens (n=17) only possessed one queen and we are taking this as evidence of monogyny in *Nylanderia parvula*. A total of 43 colonies were excavated and used for population census data collection. Among the 43 colonies, the average number of adult *Nylanderia parvula* reproductives (alate queens and males) found in colonies was: 15.4 (±23.3) for May, 0 for June, 6.1 (±4.1) for July, and 20.4 (±11.77) for September (Fig. 12). Compared to the number of alates, the total brood (larvae and pupae combined) within colonies shows the opposite trend (Figs 12, 13). Counts were low in May (37 ±134.2) and September (15.8 ±209.6), moderate in July (177.6 ±65.2), and at the highest in June (722.7 ±116.1). *Nylanderia parvula* reproductive pupae were only found in July, and *Nylanderia deceptrix* reproductive pupae were only observed in July as well.

#### Flight and dispersal.


**Figure 14.**
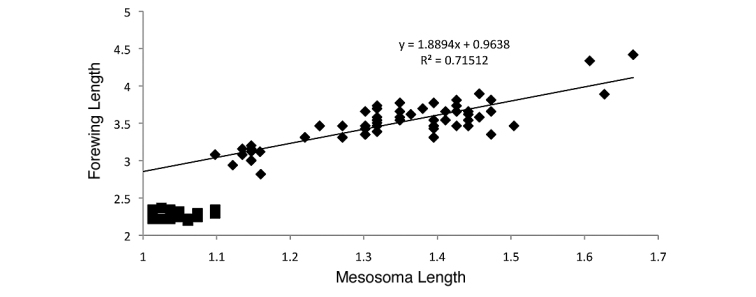
Scatter plot displaying mesosoma length (=Weber’s length) to forewing length and trendline fitting non-parasitic Nearctic *Nylanderia*: *Nylanderia arenivaga*, *Nylanderia austroccidua*, *Nylanderia concinna*, *Nylanderia faisonensis*, *Nylanderia parvula*, *Nylanderia phantasma*, *Nylanderia querna*, *Nylanderia terricola*, *Nylanderia vividula*, and *Nylanderia wojciki* (diamonds) with added *Nylanderia deceptrix* data (squares, not part of trendline data).

 Forewing length (FWL) measurements were used along with Weber’s Length to determine a ratio of forewing to Weber’s length to examine if the wings of *Nylanderia deceptrix* were smaller in proportion to *Nylanderia parvula*. The FWL:WL for *Nylanderia parvula* ranged from 2.27–2.59, with an average of 2.47 (±0.018), and for *Nylanderia deceptrix* the ration ranged from 2.07–2.31, averaging 2.18 (±0.014). Comparing the averages using a Student’s t-test, the difference between the two was significant (P<0.00001, t=12.59 for a two-tailed test), meaning the wings of *Nylanderia deceptrix* were smaller in proportion to Weber’s length compared to *Nylanderia parvula*. When examining the scatter plot of Weber’s length to forewing length of all the *Nylanderia* species used (see material and methods for list), *Nylanderia deceptrix* falls well below the trendline created from the data of the other species (Fig. 14). The R^2^ value of the trendline was significant with a P-value<0.00001 (F=98.12), indicating a true relationship between forewing length and Weber’s length for the non-obligately socially parasitic Nearctic *Nylanderia* species.

Both *Nylanderia deceptrix* and *Nylanderia parvula* queens were allowed to climb to the top of a pencil to see if they would use it as a location to take off and fly from. Five *Nylanderia parvula* queens were tested and each of them flew off of the pencil tip within two trials, however, none of the five *Nylanderia deceptrix* flew off of the pencil tip after five trials for each individual. In the lab, attempts at dropping two *Nylanderia deceptrix* queens over a white surface to provoke flight while freefalling were conducted, but neither of them flew. As *Nylanderia deceptrix* individuals were hard to collect and maintain in a laboratory setting; only two drop trials were done per individual to avoid harming or losing individuals.

#### Aggression.

The aggression tests between workers, the pairing of both *Nylanderia parvula* workers from not parasitized colonies had an average score of 2.5 (n=6, range 1–3), from one parasitized and one not parasitized colony averaged 4.4 (n=5, range 3–5), and from both parasitized colonies averaged 2.67 (n=3, range 1–4) (here colony is always referring to *Nylanderia parvula* colonies). Pairings with an *Nylanderia deceptrix* queen and a *Nylanderia parvula* worker from a not parasitized colony had an average of 5 (n=2), and with a *Nylanderia parvula* worker from a parasitized colony averaged 1 (n=2). Also, the average aggression between a *Nylanderia deceptrix* queen and a *Nylanderia parvula* queen was 1.88 (n=16, range 1–3). Introduction tests placing a *Nylanderia parvula* worker from a parasitized colony into another parasitized colony had an average score of 3.33 (n=3, range 2–5), and introducing a *Nylanderia deceptrix* queen to an already parasitized colony averaged 2.33 (n=3, range 1–5). Two of the three *Nylanderia deceptrix* introductions into an already parasitized colony resulted in acceptance of the queen into the new colony (score=1), while the third was attacked and rejected (score=5). When taking *Nylanderia deceptrix* queens or parasitized colony workers and introducing them to not parasitized colonies, the average score was 5 for each case (n=4 and 5, respectively). Similarly, workers from not parasitized colonies introduced to a parasitized colony resulted in an average score of 4.75 (n=4). The final set of introductions involved taking a *Nylanderia parvula* worker from a not parasitized colony and introducing her to another not parasitized colony. The resulting average score for that case was 4.8 (n=5, range 4–5). See Table 1 for all the aggression and introduction test average scores.

**Table 1.** Average Aggression scores (see text for details) for aggression and introduction tests (n=sample size). W/W-Colony = *Nylanderia parvula* worker to *Nylanderia parvula* worker; Dec/W-Colony = *Nylanderia deceptrix* queen to *Nylanderia parvula* worker; Dec/Queen = *Nylanderia deceptrix* queen to *Nylanderia parvula* queen.

	W/W-Colony (N)	Dec/W-Colony (N)	Dec/Queen (N)
**Parasitized to Parasitized**			
*Solo Aggression*	2.67 (3)	1 (2)	---
*Introduction Test*	3.33 (3)	2.33 (3)	---
**Parasitized to Not Parasitized**			
*Solo Aggression*	4.4 (5)	5 (2)	1.88 (16)
*Introduction Test*	5 (5)	5 (4)	---
**Not Parasitized to Not Parasitized**			
*Solo Aggression*	2.5 (6)	---	---
*Introduction Test*	4.8 (5)	---	---
**Not Parasitized to Parasitized**			
*Introduction Test*	4.75 (4)	---	---

## Discussion

The data collected about the biology and natural history of *Nylanderia deceptrix* indicates it is an obligate social parasite of *Nylanderia parvula*. *Nylanderia deceptrix* has not been observed to produce a worker caste (we observed no features among the thousands of *Nylanderia parvula* workers examined that would indicate *Nylanderia deceptrix* workers were present; i.e. all conformed to the expected worker morphology of *Nylanderia parvula*), it is functionally polygynous, host-queen tolerant, and has only been found within colonies of *Nylanderia parvula*. The intermediate size of *Nylanderia deceptrix* queens between that of *Nylanderia parvula* workers and queens is a morphological indication of its obligate social parasite status, as obligately socially parasitic ants are smaller than their host queens (Buschinger 2009). They are also often the size of their host workers but that is not the case with *Nylanderia deceptrix*. The males of *Nylanderia deceptrix* have highly reduced wings and cannot fly, a trait seen in several obligate social parasites such as *Anergates atratulus* (Schenck, 1852), *Pheidole inquilina* (Wheeler, 1903), *Plagiolepis xene* (Stärcke, 1936), and *Pogonomyrmex colei* (Snelling, 1981).

*Nylanderia deceptrix* seems to have a much more restricted range than its host and resides in an area with a high density of host colonies, traits that appear to be common among obligately socially parasitic species (Heinze 1989, Nonacs and Tobin 1992, Savolainen and Vepsäläinen 2003). The likelihood of a restricted range is enhanced by the fact *Nylanderia deceptrix* queens either have very poor flight capability, or do not fly at all, coupled with the fact that males have very small, non-functional wings. The incidence of colonies parasitized with *Nylanderia deceptrix* supports this as well, since all the colonies were clustered in close proximity to one another. The presumed poor flight capability coupled with the observed clustering of parasitized colonies leads us to suspect that *Nylanderia deceptrix* disperses by walking to nearby host colonies.

Once at a host colony, the data presented here suggest it is difficult for *Nylanderia deceptrix* to become established in a *Nylanderia parvula* colony if that colony does not already possess *Nylanderia deceptrix* queens. Our aggression data shows that *Nylanderia parvula* workers act very aggressively towards any individual (*Nylanderia parvula* worker or *Nylanderia deceptrix* queens) from a colony already parasitized with *Nylanderia deceptrix*. Comparatively, *Nylanderia parvula* displays lower aggression towards *Nylanderia parvula* workers from colonies not parasitized with *Nylanderia deceptrix*. Our data suggest that *Nylanderia parvula* can detect some kind of cue that indicates whether a *Nylanderia parvula* worker has had contact with *Nylanderia deceptrix*, resulting in the observed higher level of aggression. The reason for this high aggression towards individuals from parasitized colonies is still unknown, but it could be the result of *Nylanderia deceptrix* actually having a significant fitness cost to the *Nylanderia parvula* colonies. However, our data was not able to identify any significant fitness cost to *Nylanderia parvula* colonies.

Conversely, when an individual from a colony containing *Nylanderia deceptrix* encounters an individual from a different colony that also contains *Nylanderia deceptrix*, the aggression that results is noticeably lower. This suggests that *Nylanderia deceptrix* is influencing the amount of aggressive behavior displayed by *Nylanderia parvula*. This decrease in aggression could also be responsible for acceptance of *Nylanderia deceptrix* queens from one colony into another foreign colony already containing *Nylanderia deceptrix*. Although the mechanism and cause of acceptance for foreign *Nylanderia deceptrix* queens has not been determined, it seems likely that a contributing cause is a general decrease in aggression, a disruption in recognizing foreign individuals, or *Nylanderia deceptrix* having the ability to somehow not be recognized as a foreign individual. Overall the parasitism rate of *Nylanderia deceptrix* within *Nylanderia parvula* colonies was seemingly low at 2.5%, but it is comparable to that of several other obligate social parasites. Examples include: *Acromyrmex charruanus* at 2% (Rabeling et al. 2015), *Leptothorax wilsoni* at 1.9% (Heinze 1989), and *Cataglyphis hannae* at less than 1% (Agosti 1994). But not all obligate social parasites have such low parasitism rates. Study of *Vollenhovia nipponica* found it in over 56% of the host colonies sampled (Kinomura and Yamauchi 1992).

Our field and lab observations confirm that *Nylanderia deceptrix* is an obligate social parasite, the first known within the genus. An important next step will be to examine the phylogenetic position of *Nylanderia deceptrix* among the Nearctic *Nylanderia*, especially to see how closely related or not it is to *Nylanderia parvula* and to test whether or not a strict or loose sense Emery’s Rule applies in this example of obligate social parasitism.

## Supplementary Material

XML Treatment for Nylanderia deceptrix
